# Comparing Barriers and Facilitators to Interacting with Nature among Individuals with Different Dietary Behaviors: A Mixed Methods Study

**DOI:** 10.1016/j.tjnut.2026.101434

**Published:** 2026-02-23

**Authors:** Dahlia Stott, DeAndra Forde, Rebecca Ippolito, Jonathan M Deutsch, Mara Z Vitolins, Michael Bruneau, Jennifer A Nasser, Brandy-Joe Milliron

**Affiliations:** 1 Department of Health Sciences, College of Nursing and Health Professions, Drexel University, Philadelphia, PA, USA; 2 Department of Epidemiology and Prevention, Wake Forest University School of Medicine, Winston-Salen, NC, USA

**Keywords:** time in nature, barrier, facilitator, factor, dietary behavior, diet quality, sustainable diet

## Abstract

**Background:**

Interacting with nature is associated with positive health behaviors, including dietary choices. Understanding barriers and facilitators to interacting with nature within the context of dietary behaviors can guide development of effective interventions to promote healthy diets.

**Objectives:**

To compare barriers and facilitators to interacting with nature among individuals with higher and lower diet quality and sustainable eating behaviors.

**Methods:**

In this explanatory sequential mixed methods study, adults (*n* = 300) reported how often barriers kept them from interacting with nature. Diet quality (Healthy Eating Index-2020, range: 0‒100) and sustainable eating behavior (EAT-Lancet index, range: 0‒42) scores were determined from Diet History Questionnaire II data. A purposeful sample of participants (*n* = 30) was interviewed to compare barriers and facilitators; half the interviewees had higher diet scores (≥62.87, ≥24.00), and half had lower scores (<62.87, <24.00). Pearson’s χ^2^ tests examined whether diet scores and barriers to interacting with nature were related, and qualitative data were thematically analyzed.

**Results:**

The most frequently reported barriers to interacting with nature in the survey were hot weather (36.67%), lack of time (36.00%), and preference for indoor activities (27.67%). The mean (SD) Healthy Eating Index-2020 score was 62.87 (9.85), and the median (IQR) EAT-Lancet index score was 24.00 (5.00). Survey participants with higher diet scores less frequently reported barriers to interacting with nature. In the interviews, barriers and facilitators were identified: health, weather, time, distance, and social engagement. Interviewees with higher diet scores more frequently described nearby nature as a facilitator. Those with lower diet scores often described facilitators like socializing, having fun, and pursuing positive mental health, whereas poor weather was a deterrent.

**Conclusions:**

Findings suggest that individuals with better dietary behaviors experience fewer barriers to interacting with nature. Identifying modifiable barriers and facilitators can inform the design of interventions that integrate nature-based strategies to promote healthy and sustainable eating.

## Introduction

Consuming a healthy diet is important to promote health and decrease the risk of developing non-communicable diseases such as type 2 diabetes, cardiovascular disease, and cancer [1,2]. Despite these benefits to health, the average American does not consume a diet that aligns with the Dietary Guidelines for Americans [3]. Dietary behaviors are influenced by a variety of factors and may be impacted by where people spend their time, including their time in nature [4]. Nature can be defined as “areas containing elements of living systems that include plants … across a range of scales and degrees of human management, from a small urban park through to relatively ‘pristine wilderness’” [5].

By using a One Health paradigm, which emphasizes that the health of people is linked to the environment around them [6,7], we may be able to promote healthy dietary behaviors through increasing time in places that promote health. Researchers have demonstrated that if all adults spent ≥30 min in nature each week, cases of depression and high blood pressure would decrease by nearly 10% [8]. In addition, reductions in anxiety, stress, cortisol, and heart rate have been associated with greater exposure to nature [[9], [10], [11], [12], [13]]. Interacting with nature may also promote healthy behaviors, including greater engagement in physical activity and improvements in sleep quality and duration [[14], [15], [16], [17]].

The relationship between interactions with nature and dietary behaviors is an emerging field of study. Recently, the authors of 2 intervention studies reported that after viewing pictures of nature, participants chose to eat healthier food entrées, reported an increase in desire to consume vegetables, and reported a decrease in desire to consume snacks [18,19]. In 1 of these studies, participants who walked in nature chose to eat healthy foods, compared with participants who walked in an urban area [19]. In a convergent mixed methods study, our team identified that there are connections between interacting with nature and improved dietary behaviors, with an individual’s connection to nature playing a pivotal role [20]. We have also investigated the relationship between interactions with nature and dietary intake and found that interacting with nature was positively associated with diet quality and sustainable eating patterns [4]. Although explanatory mechanisms are still being explored, our work suggests that a person’s connection to nature and mental health may explain how interacting with nature influences dietary intake [4].

These formative research studies suggest that nature-based experiences and interventions may be used to promote dietary behaviors. By being aware of and understanding barriers and facilitators to interacting with nature within the context of dietary behaviors, practitioners can develop effective interventions that use time in nature to improve dietary behaviors. Further, by promoting a dietary pattern that is beneficial for human and planetary health and time in nature, people may recognize the importance of living within planetary boundaries and move toward achieving socioecological equilibrium [21]. The purpose of this study was to identify and compare barriers and facilitators to interacting with nature between individuals with higher and lower diet quality and sustainable eating behaviors. By using a mixed methods approach, barriers and facilitators to interacting with nature can be compared across groups and sources of data, which can provide actionable recommendations to promote time in nature and, therefore, dietary behaviors.

## Methods

**FIGURE 1:**
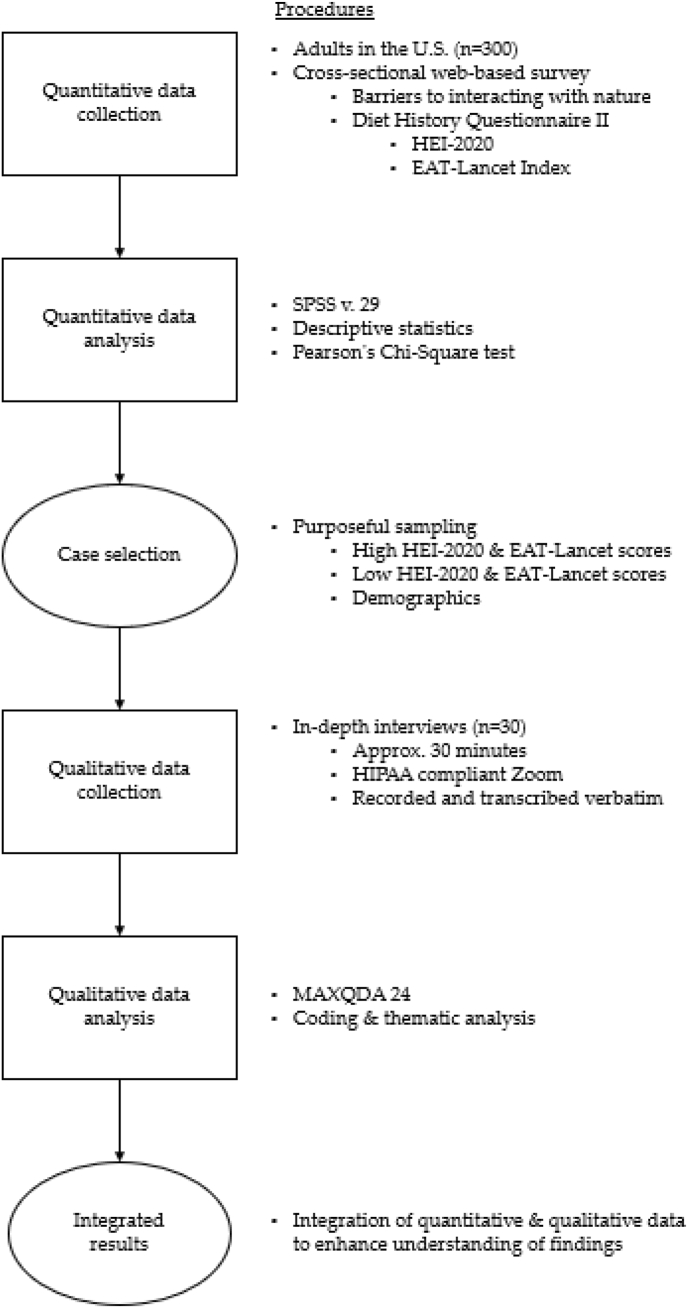
Explanatory sequential mixed methods study. HEI-2020, Healthy Eating Index-2020; U.S., United States; HIPAA, Health Insurance Portability and Accountability Act; SPSS, Statistical Package for the Social Sciences.

This is a sub-analysis of an explanatory sequential mixed methods study that investigated the relationships among interactions with nature and dietary behaviors (Figure 1) [4]. Quantitative data were collected from September 2023 to November 2023 and were used to determine the participants sampled for the qualitative phase. Qualitative data were collected from May 2024 to June 2024. Both sets of data were connected through purposeful sampling in the qualitative phase by diet behaviors measured in the quantitative phase. A mixed methods study design was employed to gain a deeper understanding of barriers and facilitators to interacting with nature, as combining quantitative measures with personal experiences can provide a more comprehensive understanding than either form of data alone [22]. This study was deemed exempt by the institutional review board at Drexel University (protocol number: 2302009742).

### Participants

Using ResearchMatch and Build Clinical as recruitment partners, adults residing across the United States were recruited to participate in an online survey. ResearchMatch is a registry that connects individuals who are interested in participating in research studies with researchers via email. Build Clinical runs digital advertisement campaigns to recruit for research studies. Participants were eligible for the study if they were ≥18 y old, lived in the United States, had at least a high school education or equivalent, had access to a device and internet, and spoke English. After being recruited for the research study, participants reviewed the consent form and gave consent online in proxy to written informed consent before starting the study.

In the qualitative phase, a purposeful sample of participants was interviewed: half of the interviewees had higher diet scores, and half had lower diet scores, relative to the sample. Participants in the qualitative phase were also purposely sampled by their demographics (i.e., age, race, and gender). This sampling strategy was employed to ensure that a diverse set of perspectives could be obtained and to allow for comparing barriers and facilitators during data integration.

### Quantitative phase

Once participants gave consent, they completed an online survey on Qualtrics, after which they were provided $10 in compensation. This survey was piloted in a previous study [20]. In this survey, participants reported how often certain barriers kept them from interacting with nature on a 4-item Likert scale [8]. The options were: “Never,” “Sometimes,” “Often,” and “Most of the time.” Given the low response to “Most of the time” (0‒11%), “Often” and “Most of the time” responses were combined for analysis.

Participants also completed the Diet History Questionnaire II, a semi-quantitative food frequency questionnaire that asked participants about their dietary intake over the previous month [23]. Diet quality [Healthy Eating Index-2020 (HEI-2020)] and sustainable eating behavior (EAT-Lancet index) scores were calculated from this data. HEI-2020 measures adherence to the Dietary Guidelines for Americans 2020‒2025 edition, with greater scores (0‒100) indicating greater adherence [24]. The EAT-Lancet index measures adherence to the EAT-Lancet reference diet that was developed to address the growing need for dietary recommendations to promote both human and planetary health [25,26]. A greater EAT-Lancet index score (0‒42) indicates greater adherence to the reference diet.

### Qualitative phase

The interviews focused on understanding barriers and facilitators to interacting with nature, were led by a qualitative researcher (DS), used a semi-structured interview guide, and used a descriptive, phenomenological approach [27]. The interview guide was developed by the lead and senior authors (DS and B-JM) and reviewed independently by other authors (JMD and MZV) before qualitative data collection began. Questions that elicited the barriers and facilitators to interacting with nature were: “I noticed in your survey that you spend time at [specific nature spaces (i.e., beaches and parks)]. What are some of the reasons why you spend time in these locations?” “Are there obstacles or challenges that prevent you from spending more time in nature? What prevents you from spending time in nature?” “Is there anything else you can share about things that make it harder or easier to spend time in nature?” The interviews were conducted on Health Insurance Portability and Accountability Act compliant Zoom, lasted ∼30 min, were recorded, and transcribed verbatim. Field notes were also taken. Participants were provided with $15 for their time.

### Data analysis

In the quantitative phase, IBM SPSS Statistics versions 29 and 30 were used to generate descriptive statistics. Pearson’s χ^2^ test was used to assess if barriers to interacting with nature and diet score are related. In the qualitative phase, researchers used a thematic analysis with an inductive approach in MAXQDA 24 [28]. Three researchers (DS, DF, and RI) with qualitative experience first open-coded 4 interviews; after which, a code book was developed and iteratively refined. The rest of the interviews were double-coded, and discrepancies between the codings were discussed and resolved. Once all interviews were coded, the researchers identified factors that represented barriers and facilitators to interacting with nature. During analysis, the researchers determined that data saturation was reached. During data integration, we compared the data sets to observe congruence and divergence. Joint displays were created to compare barriers and facilitators to interacting with nature [29].

### Enhancing validity and credibility

Steps were taken throughout the study to enhance validity and credibility. In the quantitative phase, using validated instruments to measure dietary intake ensured that there was internal validity, reliability, and generalizability [30]. Having inclusion and exclusion criteria also helped ensure generalizability [30]. In the qualitative phase, credibility and confirmability were ensured by triangulating the 2 sets of data [31]. Confirmability was established by recording all the research activities [31]. Having multiple researchers analyze the qualitative data established dependability, and transferability was ensured by purposeful sampling [31].

## Results

### Quantitative results

**FIGURE 2:**
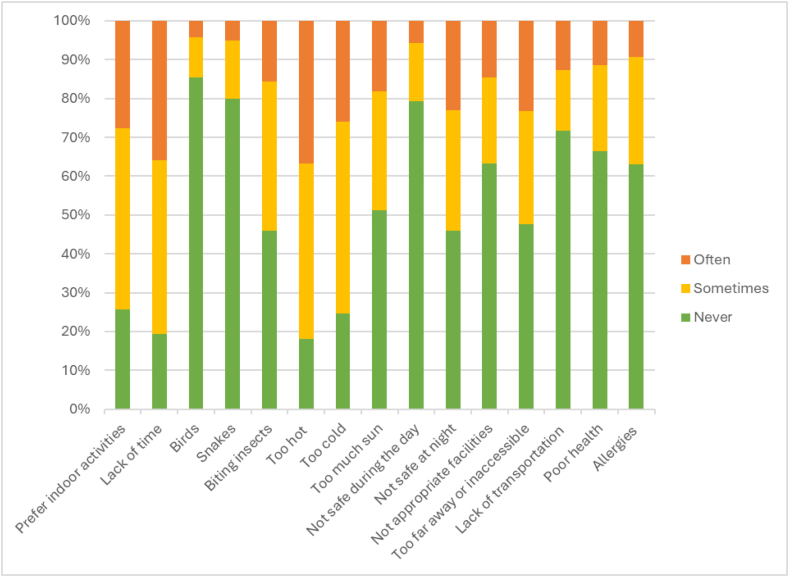
Frequency of barriers to interacting with nature.

Three hundred adults completed the survey. The 3 most frequently reported barriers to interacting with nature included the weather being too hot (36.67%), a lack of time (36.00%), and a preference for indoor activities (27.67%). The least reported barriers included birds (14.67%), snakes (20.00%), and lack of safety during the day (20.67%). Supplemental Table 1 and Figure 2 display the frequency of barriers reported.

The mean (SD) HEI-2020 score was 62.87 (9.85), and the median (IQR) EAT-Lancet index score was 24.00 (5.00). One hundred participants had higher HEI-2020 (≥62.87) and EAT-Lancet index scores (≥24.00), whereas 112 had lower scores (<62.87 and <24.00, respectively). There were significant associations between being categorized as having a higher or lower diet score and certain barriers to interacting with nature. These specific barriers include: preference for indoor activities [χ^2^(2) = 8.72, *P* = 0.01], lack of time [χ^2^(2) = 7.13, *P* = 0.03], pigeons or other birds [χ^2^(2) = 8.77, *P* = 0.01], snakes [χ^2^(2) = 7.68, *P* = 0.02], biting insects [χ^2^(2) = 8.91, *P* = 0.01], not appropriate facilities [χ^2^(2) = 7.46, *P* = 0.02], facilities are too far away or inaccessible [χ^2^(2) = 7.97, *P* = 0.02], a lack of transportation [χ^2^ 2) = 7.17, *P* = 0.01], and allergies [χ^2^(2) = 6.57, *P* = 0.04]. Individuals in the higher diet score group less often reported barriers to interacting with nature. Frequency of reported barriers by diet score categories and Pearson χ^2^ results are presented in Table 1.

**TABLE 1:** Frequency of barriers to interacting with nature by diet score categories^1^

	Lower diet scores (*n* = 112)			Higher diet scores (*n* = 100)			*χ*^*2*^	*P* value
	Never	Sometimes	Often	Never	Sometimes	Often		
Prefer indoor activities	19 (16.96)	55 (49.10)	38 (33.93)	32 (32.00)	48 (48.00)	20 (20.00)	8.72	0.01
Lack of time	14 (12.50)	53 (47.32)	45 (40.18)	27 (27.00)	40 (40.00)	33 (33.00)	7.13	0.03
Pigeons or other birds	90 (80.36)	16 (14.29)	6 (5.36)	94 (94.00)	5 (5.00)	1 (1.00)	8.77	0.01
Snakes	83 (74.11)	23 (20.54)	6 (5.36)	89 (89.00)	9 (9.00)	2 (2.00)	7.68	0.02
Biting insects	40 (35.71)	48 (42.86)	24 (21.43)	54 (54.00)	36 (36.00)	10 (10.00)	8.91	0.01
Too hot	15 (13.39)	47 (41.96)	50 (44.64)	24 (24.00)	40 (40.00)	36 (36.00)	4.25	0.12
Too cold	24 (21.43)	54 (48.21)	34 (30.40)	30 (30.00)	45 (45.00)	25 (25.00)	2.19	0.34
Worried about too much sun	53 (47.32)	35 (31.25)	24 (21.43)	63 (63.00)	25 (25.00)	12 (12.00)	5.87	0.05
Not safe during the day	84 (75.00)	23 (20.54)	5 (4.46)	86 (86.00)	13 (13.00)	1 (1.00)	4.80	0.09
Not safe at night	46 (41.11)	39 (34.82)	27 (24.11)	54 (54.00)	28 (28.00)	18 (18.00)	3.58	0.17
Not appropriate facilities	64 (57.14)	27 (24.11)	21 (18.75)	75 (75.00)	14 (14.00)	11 (11.00)	7.46	0.02
Facilities are too far away or inaccessible	46 (41.07)	32 (28.57)	34 (30.36)	60 (60.00)	22 (22.00)	18 (18.00)	7.97	0.02
A lack of transportation	74 (66.07)	15 (13.39)	23 (20.54)	80 (80.00)	12 (12.00)	8 (8.00)	7.17	0.03
Poor health	74 (66.07)	23 (20.54)	15 (13.39)	71 (71.00)	20 (20.00)	9 (9.00)	1.10	0.58
Allergies	63 (56.25)	36 (32.14)	13 (11.61)	71 (71.00)	25 (25.00)	4 (4.00)	6.57	0.04

1 Frequency is presented as *n* (%).

### Qualitative results

Thirty of the 300 participants were interviewed to explore barriers and facilitators to interacting with nature. Fifteen of these participants had higher diet scores, and 15 had lower scores. Factors that influenced participants’ time in nature (barriers and facilitators) included health, weather, distance, time, and social engagement. Supplemental Table 2 displays the factors, codes, and exemplary quotes.

#### Health

Multiple participants described that being in nature positively influenced their mental health. For example, P009 said, *“If I’m stressed out a lot, I can use the environment in nature to help me, and at other times it can… reverse where I’m just too in my head or stressed out*.*”* Nature was described multiple times as being peaceful, and having these moments allowed participants to experience awe. For P032, experiencing peace while in nature, let them reflect on the beauty of the larger picture. *“It’s just very calming, the noises. It’s like a different type of quiet, for the most part. And then just admiring, like, oh, this all occurred and was here years before I existed. It’s nice,”* P226 said.

Nature was a place where physical activity, such as hiking, biking, and walking, could take place. P074 and P321 talked about how they lived in an urban area but made an effort to run outdoors and in nature. *“Part of it is just exercise and getting vitamin D rather than being in the house or in the gym … I want to get out and just get the sun ray*s,*”* said P074. One participant recognized the added benefit of exercising in nature. P045 said they had a goal of biking a certain number of miles each week to reduce their blood pressure. Later in the interview, they referenced that studies have shown that being in nature reduces blood pressure, and they felt that was true in their life.

Participants described how health conditions such as allergies, injuries, and limited mobility kept them from being in nature. Playing basketball was P204’s main activity in nature, but after sustaining a torn rotator cuff, they no longer went out into nature as often as they once had. Even though they loved being in nature, P050 and their partner had physical limitations. *“There aren’t really a lot of nature spaces that are designed for low mobility,”* P050 said.

#### Weather

Weather patterns and seasons were influential in how often participants went outdoors. For most participants, mild weather prompted them to spend more time in nature. P072 said, *“If the weather is forecast to be nice, I make a concerted effort to get out.”*

Hot and cold weather deterred participants from going out into nature. Participants described that in hot months, warm temperatures and humidity kept them indoors. *“I mean, it’s a lot more humid here than I’m used to, so we just feel gross after a while,”* said P049. P072 recognized that they could enjoy nature in the summer, if they could get outside early enough, *“It’s just like blazing hot if you don’t get out in the morning.”* During the colder months of the year, participants described that it was harder to go outside. P031 was particularly hindered by the weather because of their health and how their body responds to weather conditions, *“When it’s raining or cold, my body don’t want to move. It hurts. I have to force myself sometimes to move.”* Other poor aspects of weather or seasons, such as pollution and bugs, were also deterrents. *“I do live in a very urban environment where I’m sure there’s a lot of smog, and just pollution,”* said P321. P245 is a physician and described that they saw diseases related to ticks in their work; *“The pathology of the things that we’re seeing in nature right now that really makes me not like nature as much as I did as a kid.”*

#### Distance

Several participants lived in an urban area and described how difficult it was for them to get to a place with nature. Not being able to drive was a barrier for P114 to get into nature, given that they didn’t live near any natural landscapes:

“There [are] certain buses that go to the park, but then you have to find a time that you can get there, and then obviously you have to find the time to leave. Because if you leave when the bus isn’t there, it’s really hard to get home or anywhere else.”

Having nature around their home and in their neighborhoods facilitated participants to spend more time in nature. Looking out through windows was 1 way that participants were able to appreciate nature. *“I’ve got [a] wall of windows in my kitchen that overlooks …[an] outdoor, common area lake. … I love looking out the window,”* said P277. Parks and other areas of nature near the home were often used by participants. P099 took their children to a sports complex multiple times per week, which has natural paths so they would *“just go for a walk … in the hills around there.”*

#### Time

Having free time made it easier and motivated participants to spend time in nature. There are greenspaces around P004’s office, and they described, *“When there’s breaks, I’ll just walk around and try to have a quiet time and look outside and then come back to the office.”* Multiple participants described the weekend as the time that they spent outdoors because they did not have any other responsibilities. *“Four days a week, I feel like I gotta do everything that somebody tells me. But Friday, Saturday, Sunday is … just fresh air,”* said P031. Being in nature also allowed people to have time for themselves. P245 was able to go into nature by themself and found the experience to be *“calming.”* P329 liked that being in nature allowed for *“personal [time] with me and with my thoughts.”* During vacations, participants made time to be outdoors and take part in activities that they don’t often do at home. For example, P099 described that they are *“typically doing a lot of outdoor stuff when [they are] on vacation.”*

Participants described they do not have as much time to go into nature as they would like. Work was the time-related barrier most often discussed. P256 was working and applying to school; although they recognized that being in nature may promote their mental state, they simply did not have time. *“I’d rather be outside, and like, I think being outside kind of relieves some of the stress, but I just don’t have time to go outside.”* Family also kept some participants from being out in nature. P074 described,

“Having 2 kids, I have a lot less flexibility. I guess pre-kids, like it was easier to justify taking 30 min to just go running or something during my lunch break, or just get the dog out for an extra walk midday.”

#### Social engagement

Being outdoors and in nature was a good time for our participants to connect with their family and friends. P049 described, *“[Being in nature] keeps me off my phone. When my wife and I spend time outside, I feel like that maybe encourages a little bit more interpersonal time*.*”* Fostering an appreciation for nature in their children was 1 of the motivators for being outdoors. *“I try to grow things on my own property, which is not a big production, but it’s more just teaching my kids … where food comes from a little bit,”* said P074.

Being with others motivated multiple participants to be in nature. P130 said, *“I like having a fun time. … [I] like to relax and sit and have fun and chat with people.”* Connecting with their friends motivated P241 to be outdoors, *“I also enjoy spending time with nature with my friends. I feel like there’s a kind of union of connectedness, and everybody just kind of trying to feel good.”* Having animals promoted participants to go out into nature more often, even for those who reported more barriers to going outdoors. *“My main interaction [with nature] is just taking my dogs out,”* said P226. Even though P049 spent quite a bit of time outdoors, they still saw their dog as important for consistency: *“The dog keeps us honest in terms of at least getting outside.”*

### Integrated results

We identified how often barriers and facilitators were discussed within the 2 groups of participants and compared these across groups. Among interviewees who had higher diet scores, time for other commitments, poor weather, and poor health, specifically in mobility limitations, were the most often reported barriers to interacting with nature. The most often reported facilitators were nature being close by, family, mental health benefits, and doing physical activity. Interviewees in the lower diet score group most often reported poor weather, a general lack of time, and poor health as barriers to interacting with nature. Nature being close by, social and mental health benefits were the most often reported facilitators to interacting with nature between the lower diet score group.

Differences were observed in how often participants talked about the factors that impact whether they interact with nature. Compared with participants in the lower diet score group, participants in the higher diet score group spoke more often about how they recognized and went to nature that was close by. Participants in the lower diet group more often spoke about facilitators, including socializing, having fun, and pursuing positive mental health, compared with the higher diet score group. Poor weather was more often reported between interviewees in the lower diet score group, but in the quantitative data, there were no significant differences in the frequency of weather being too hot or too cold among participants with higher and lower diet scores. Exemplar cases are presented in Table 2.

**TABLE 2:** Joint display of exemplar participants’ quantitative measures, quotes, and mixed methods interpretation

	Quantitative results	Exemplar quotes	Mixed methods interpretation
P092	HEI-2020 : 74.52 EAT-Lancet index: 36	“[My partner and I do] a combination of things. Quite a bit of hiking. … So, we go down to [a local] park a lot and do hiking there. … we do a lot of camping … we do some kayaking … as we get more used to the city and getting to the trails and stuff, but we’ll do bicycling. So those are the primary things: camping, hiking, kayaking, and bicycling.” “The building we’re in, the apartment building, has a rooftop area, a natural area with plantings, and we frequently go up there, and it’s a green roof. It’s planted with sedum all through. And from there we can look out over the [local] park in [state]. And there’s lots of parks around. So, we have a good option of, of going and seeing stuff there.”	This participant has higher diet scores and goes out into nature often. They had recently moved to an urban city, which made it more difficult than usual to go into nature, but they still found nature areas around their home to frequent. They also went on longer trips in nature to go camping, hiking, and kayaking.
P163	HEI-2020: 45.53 EAT-Lancet index: 16	“My hometown is near beaches. So, like whenever I come to visit, I like later this month, I think my plans are just to go to the beaches and stuff like that.” “I’m very anti going outside because of the cold. I would say ideally, the most optimal time is either in the spring or the fall. I would say I’m not a huge fan of the heat.” “So, my hospital also has a view of the river and likes the greenery, and so it trails. So, like just seeing the weather, seeing like everyone outside. It’s definitely why I have a desire to go outside instead of being crammed up.”	This participant has lower diet scores and does not spend a lot of time in nature. Other commitments, such as their job and schooling, kept them from going out into nature. Although they recognized that nature was close to them, they infrequently had a desire to spend time there. They spent more time in nature when they went back to their hometown, but they were deterred from going outdoors when there was poor weather. Pleasant weather and a desire to be with others prompted this participant to wish to be in nature.

Abbreviations: HEI-2020, Healthy Eating Index-2020; P092, P163, participants.

There were similarities and differences between the quantitative and qualitative data. In the survey, 45.33% of participants reported that hot weather “Sometimes” kept them from being outdoors, whereas 36.67% reported that this “Often” kept them from being outdoors. Poor weather was also consistently reported as a barrier in the interviews. Only 19.33% of participants reported that a lack of time was never a barrier to interacting with nature, and this barrier was also reported in the interviews.

Although 74.33% of participants reported a preference for indoor activities, keeping them from going into nature, “Sometimes” or “Often,” only 4 interviewees mentioned a preference for other activities, which were not described in detail. More than half the participants reported biting insects as a barrier in the survey, but only 6 interviewees conveyed that bugs such as mosquitoes and ticks drove them indoors. In comparison, poor health and allergies were some of the least reported barriers (33.67% and 37.00%, respectively), but were more often reported in the interviews.

## Discussion

In this mixed methods study, we identified that health, weather, distance, time, and social engagement are both barriers and facilitators to interacting with nature. We also compared barriers and facilitators between interviewees with higher and lower diet scores. We found that those with higher diet scores more often spoke about using nature that was nearby, whereas those with lower diet scores more often discussed socializing, having fun, and positive mental health as facilitators and poor weather as a barrier. Furthermore, the integrated results highlight that there were similarities and differences in barriers reported in the surveys and interviews. In both, poor weather was reported as a barrier, whereas preferences in other activities and biting insects were more often reported in the survey.

Some interviewees who described weather as a barrier to interacting with nature still went outdoors, even if their time in nature was limited. The ways that participants spent time in nature and the aspects that they enjoyed about nature during periods of poor weather varied. Some participants talked about being in nature during hot days by spending time around bodies of water and going into nature in the mornings. During the winter, participants enjoyed that there were less insects. Participants also discussed that the beauty of nature can be enjoyed in each season.

When describing distance as a barrier to spending time in nature, participants were mainly thinking about larger nature spaces that are typically farther away from where people may live (i.e., woods, mountains, lakes, beaches, and hiking trails). In comparison, many participants discussed the nature near where they lived, including yards, neighborhoods, and parks. Nature doesn’t need to be far away for people to enjoy it and experience its health benefits [32]. Some participants described walking or biking to nature spaces, but others spoke about not being able to use public transit to get to nature. This suggests that different means of transportation affect the ability to interact with nature. Future work is needed to explore how utilizing different modes of transportation is related to interacting with nature, especially across urban and rural areas.

Work and family responsibilities were the primary reasons for limited time in nature [[33], [34], [35]]. Even so, some people spend time in nature during the weekends, in their yards, and outside their workplaces during short breaks. Researchers recommend that people spend between 90 min and 120 min in nature each week [8,36]. This commitment may be daunting to people, but this equates to 12‒17 min in nature each day of the week, which may be more reasonable to accommodate in a busy schedule.

Descriptions of health and social engagement as facilitators to interacting with nature highlight that nature can serve as the venue for multiple things. It was described as a place where people engaged in physical activity and a place where they could relax [[37], [38], [39], [40], [41]]. Nature can also be a location where people can spend time with family, friends, and pets, take part in community activities, and enjoy hobbies [[38], [39], [40]]. Additionally, there were some participants who were unable to spend time in nature because of mobility and physical limitations [33,37]. By identifying health-related or social experiences that make being in nature valuable and ensuring that nature spaces are accessible to all, people may be prompted to spend more time in nature.

We identified that P045 and P188 were participants who discussed several barriers to interacting with nature, but also how they overcame the barriers. Their experiences offer some recommendations as to how to promote time in nature. P045 described that they went into nature often when they biked. They had a back injury that sometimes kept them from biking, and in those days, they still walked in nature with their spouse. They worked a full-time job and visited their family on the weekends. Despite these time-related barriers, they prioritized their bike rides in nature for their health. P188 had a busy schedule, and walking their dog allowed them to spend some time in nature every day. This participant also described that during the winter, they still visited nature, even if they had less excitement about it. They lived in a large urban city; although they described large nature spaces as being far away, they frequently visited small parks near their home. The insights from these participants suggest several strategies to overcome barriers to interacting with nature. First, people can find meaningful and personal reasons to spend time in nature. Second, they can find smaller nature spaces near their home. Third, people can set apart time to be in nature, even to achieve another goal, such as exercising or walking the dog.

Although the qualitative results provide general barriers and facilitators to interacting with nature, the integrated findings provide insights into which barriers and facilitators are experienced more often by individuals with high and low diet scores for diet quality and sustainable dietary behaviors. The most discussed barriers to interacting with nature were the same for those in the higher and lower diet score group but weather was discussed as a barrier more often by the lower diet score group. Similarly, close-by nature and mental health benefits were both often reported by both groups. Nature being close by was more often discussed by the interviewees in the higher diet score group, and mental health benefits were discussed more by the interviewees in the lower diet score group.

These integrated findings suggest targeted strategies for promoting dietary intake through time in nature. First, we can identify nature spaces that are easily accessible and explore strategies for promoting engagement during adverse weather conditions or motivating people to engage with nature in a variety of conditions, not simply when the weather is “nice.” Second, we can educate people about the mental and physical health benefits of interacting with nature. Third, we should encourage individuals to spend time with others and have fun while in nature. Health care professionals, urban planners, and community organizations who are focused on One Health should be aware of these barriers and facilitators to develop effective interventions and increase motivation and adherence to the intervention. Although while delivering these future interventions, it is important to meet people where they are, as lived experiences need to be considered when eliciting meaningful behavior change.

During data integration, we observed convergence and divergence between the quantitative and qualitative data. Poor weather and lack of time were barriers to interacting with nature in both sets of data. Although more interviewees with lower diet scores described poor weather as a barrier to interacting with nature, we did not observe a difference in the frequency of poor weather as a barrier between participants with higher and lower diet scores. Notably, the survey data highlighted a higher prevalence of indoor activity preferences and insect-related concerns, whereas interviews emphasized health-related barriers such as allergies and mobility limitations. This divergence may be attributed to differences in data collection methods—structured survey prompts compared with open-ended interview responses. Ultimately, these findings highlight that mixed methods are important to give greater context and depth to research findings [22].

There are several strengths and limitations to this study. To the best of our knowledge, we are the first to compare barriers and facilitators to interacting with nature among individuals of differing behaviors. In the qualitative phase, we used purposeful sampling across diet scores and demographics to ensure that we captured a diverse set of experiences. The use of mixed methods allowed for a greater context to the barriers and facilitators than either form of data would provide alone. However, the current study is not without limitations. We prioritized assessing barriers to interacting with nature, and therefore, future studies should place equal emphasis on barriers *and* facilitators in their mixed methods analyses. Differences in data collection methods may account for some of the divergence we observed between quantitative and qualitative responses. Additionally, we recruited people with at least a high school education, which limits the generalizability of these findings.

This research informs targeted interventions that leverage environmental accessibility, social engagement, and awareness of health benefits to promote sustained nature engagement. Our findings bolster the importance of One Health by underscoring the potential role of environmental and social factors and suggest opportunities for enhanced public health and urban planning to support healthful dietary behaviors through nature-based interventions.

## Author contributions

The authors’ responsibilities were as follows – DS, B-JM: conceptualization and methodology; DS: investigation; DS, DF, RI: formal analysis; DS: writing – original draft; DF, RI, JMD, MZV, MB, JAN, B-JM: writing- review and editing; DS: visualization; B-JM: supervision; DS, JMD, B-JM: funding acquisition; and all authors: read and approved the final manuscript.

## Data availability

The data that support this article are available from the corresponding author.

## Funding

This work was supported by the Dean’s Rapid Response Relevant Grant (R3), College of Nursing and Health Professions, Drexel University, and a Summer Research Award from Drexel University.

## Conflict of interest

The authors report no conflicts of interest.
