# Supplementary material for: Comparing Barriers and Facilitators to Interacting with Nature among Individuals with Different Dietary Behaviors: A Mixed Methods Study
Source: J Nutr. 2026 Feb 23;156(4):101434. doi: 10.1016/j.tjnut.2026.101434 (PMC13084594; doi:10.1016/j.tjnut.2026.101434)
Supplement: Multimedia component 1 [file mmc1.docx]

**Supplementary material belonging to:
Comparing barriers and facilitators to interacting with nature among individuals with different dietary behaviors: A mixed methods study**

Stott D, Forde D, Ippolito R, Deutsch JM, Vitolins MZ, Bruneau Jr. M, Nasser JA, Milliron BJ

Supplemental Table 1. Frequency of barriers to interacting with nature^a^

|  | Never | Sometimes | Often |
| --- | --- | --- | --- |
| Prefer indoor activities | 77 (26) | 140 (47) | 83 (28) |
| Lack of time | 58 (19) | 134 (45) | 108 (36) |
| Pigeons or other birds | 256 (85) | 31 (10) | 13 (4) |
| Snakes | 240 (80) | 45 (15) | 15 (5) |
| Biting insects | 138 (46) | 115 (38) | 47 (16) |
| Too hot | 54 (18) | 136 (45) | 110 (37) |
| Too cold | 74 (25) | 148 (49) | 78 (26) |
| Worried about too much sun | 154 (51) | 92 (11) | 55 (18) |
| Not safe during the day | 238 (79) | 45 (15) | 17 (6) |
| Not safe at night | 138 (46) | 93 (31) | 69 (23) |
| Not appropriate facilities | 190 (63) | 66 (22) | 44 (15) |
| Facilities are too far away or inaccessible | 143 (48) | 87 (19) | 70 (23) |
| A lack of transportation | 215 (72) | 47 (16) | 38 (13) |
| Poor health | 199 (66) | 67 (22) | 34 (11) |
| Allergies | 189 (63) | 83 (28) | 28 (9) |

^a^ Represented as n(%)

| Supplemental Table 2. Factors, codes, and exemplary quotes | | |
| --- | --- | --- |
| Factor | Code | Exemplary Quotes |
| Health | Peaceful (Facilitator)  Mental Health (Facilitator)  Physical activity (Facilitator)  Mobility issues (Facilitator)  Health (Barrier) | “It was kind of one of my happy place where I kind of find comfort and peace” – P241  “And then when I do want to go out in nature, it's typically when I'm really stressed and I need, like, a break and a reset.” – P226  “So I have a seven year old, and it's really important. Mostly exercise. Honestly. Mostly exercise. It's really important for us to be some sort of an active family. So once a week we try to do something active” – P245  “I used to love to hike until my physical mobility became more impaired. And also my partner had mobility issues, so I, I do love nature. I wish I spent more time in nature. I just don't always have much opportunity.” – P250  “Mostly I think I was just about to say, I guess allergies. I've been bothered me a little bit since moving. Don't know whether it'll settle down after a little bit or not” – P049 |
| Weather | Weather (Facilitator)  Weather (Barrier) | “Temperature is the best there at that time. It's nice and crisp and cool. Very low humidity. Very bearable temperature. The bugs will still be there, you know, but, that's more tolerable than, you know, the weather. I do get allergies, but, you know, I can just take my allergy medication and, you know, go out there and spend, you know, spend almost an entire day.” – P277  “I ain't really a big fan of snow, so I ain't going out.” – P204  “I would say during winter … I'm very anti going outside because of the cold.”- P256 |
| Distance | Nature close-by (Facilitator)  Distance (Barrier)  Accessibility and Transportation (Barrier) | “The biggest thing that makes it easier, is, again, having the facilities here. I mean, that's one of the things we know that there are places to go biking. There are lots of bike paths, lots of places to go biking and even some of the places, on the streets are, are safe. … [local park], it's a huge park, and run by the National Park Service and lots of hiking trails and lots, lots available. So the options available.” – P092  “It makes it more difficult to spend time with nature if I don't have something like, available within like a short car ride or a short walk away.” – P188  “So I don't live in a very walkable neighborhood. So you have to drive everywhere. There's not a walkable park. There's my garden that I, you know, can walk to, of course, but if I want a change in scenery, you need a car.” – P350 |
| Time | Time (Facilitator)  Time (Barrier)  Other commitments (Barrier) | “A of weekends, the vibes of holidays coming, weekend holidays the rush is not so much. So a few a few more relaxed to spend time with nature at that time.” – P320  “Probably sometime I might want to spend, time in nature with my friends, and sometime they might like, okay, we don't have time, you know, to go. So sometimes I feel like a setback, you know, like a drawback.” – P241  “If I have a lot of work, and if it's, I guess bad, bad managing my time. If I procrastinate too much. Then of course, if there's a lot, a lot of traveling, with, with family and things like that, that that can be obstacle.” – P004 |
| Engagement | Social/emotional benefits & Children (Facilitators)  Being with others (Facilitator) | “I think that what makes it easier would be the environment you're in, that when you find a source of enjoyment you really like, that really sits right with you and you have pleasant memories and thoughts about that. It's something you want to enjoy more often. You want to go back. You want to be there and watching my kids and my dogs get happy and excited and that that's what really works for me. That helps me feel even more enjoyment.” – P090  “But personally, for me, I enjoy, you know, spending time in nature. But I also enjoy spending time with nature, with my friends. I feel like there's the kind of, you know, the union of connectedness and everybody just kind of trying to feel good.” – P241 |
